# Functional and patient-reported outcome versus in-hospital costs after traumatic acute subdural hematoma (t-ASDH): a neurosurgical paradox?

**DOI:** 10.1007/s00701-019-03878-5

**Published:** 2019-03-28

**Authors:** Jeroen T. J. M. van Dijck, Thomas A. van Essen, Mark D. Dijkman, Cassidy Q. B. Mostert, Suzanne Polinder, Wilco C. Peul, Godard C. W. de Ruiter

**Affiliations:** 1 Department of Neurosurgery, University Neurosurgical Center Holland (UNCH), Leiden University Medical Center & Haaglanden Medical Center & Haga Teaching Hospital, Leiden/The Hague, The Netherlands; 2 Department of Public Health, Erasmus Medical Center, Rotterdam, The Netherlands

**Keywords:** Acute subdural hematoma, Traumatic brain injury, Treatment, Patient outcome, Healthcare costs

## Abstract

**Background:**

The decision whether to operate or not in patients with a traumatic acute subdural hematoma (t-ASDH) can, in many cases, be a neurosurgical dilemma. There is a general conception that operating on severe cases leads to the survival of severely disabled patients and is associated with relatively high medical costs. There is however little information on the quality of life of patients after operation for t-ASDH, let alone on the cost-effectiveness.

**Methods:**

This study retrospectively investigated patient outcome and in-hospital costs for 108 consecutive patients with a t-ASDH. Patient outcome was assessed using the Glasgow Outcome Score (GOS) and the Traumatic Brain Injury (TBI)–specific QOLIBRI questionnaire. The in-hospital costs were calculated using the Dutch guidelines for costs calculation.

**Results:**

Out of 108 patients, 40 were classified as having sustained a mild (Glasgow Coma Scale (GCS) 13–15), 19 a moderate (GCS 9–12), and 49 a severe (GCS 3–8) TBI. As expected, mortality rates increased with higher TBI severity (23%, 47%, and 61% respectively), whereas the chance for favorable outcome (GOS 4–5) decreased (72%, 47%, and 29%). Interestingly, the mean QOLIBRI scores for survivors were quite similar between the TBI severity groups (61, 61, and 64). Healthcare consumption and in-hospital costs increased with TBI severity. In-hospital costs were relatively high (€24,980), especially after emergency surgery (€28,670) and when additional ICP monitoring was used (€36,580).

**Conclusions:**

Although this study confirms that outcome is often “unfavorable” after t-ASDH, it also shows that “favorable” outcome can be achieved, even in the most severely injured patients. In-hospital treatment costs were substantial and mainly related to TBI severity, with admission and surgery as main cost drivers. These results serve as a basis for necessary future research focusing on the value-based cost-effectiveness of surgical treatment of patients with a t-ASDH.

**Electronic supplementary material:**

The online version of this article (10.1007/s00701-019-03878-5) contains supplementary material, which is available to authorized users.

## Introduction

Traumatic brain injury (TBI) is accompanied by an acute subdural hematoma (t-ASDH) in around 10–20% of admitted TBI patients [4]. Despite neurosurgical treatment, mortality rate is high (40–60%) and outcome is often unfavorable (up to 70%) [4, 9, 21, 31]. This frequently poses an ethical dilemma for neurosurgeons, especially in the more severe cases. Neurosurgical evacuation of the hematoma, sometimes with additional decompressive craniectomy (DC), can save patients’ lives by decreasing intracranial pressure and preventing secondary edema, ischemia, and inflammatory cell death, but at the same time, it may result in the survival of severely disabled patients [16, 23]. Alternatively, early treatment-limiting decisions (TLD) reduce any chance of recovery and normally result in death [35, 50]. To assist physicians in these difficult life-or-death decisions, experts in the field have provided statements and guidelines on the preferred treatment strategies in these patients [4, 5]. However, the overall adherence to these guidelines is low, probably because the general conception is that outcome for these patients is rather “unfavorable” [6, 7, 43].

Unfortunately, in the literature, there is little information on the health-related quality of life (HRQoL) after surgical treatment of patients with a t-ASDH. Until recently, researchers used functional indicators like the Glasgow Outcome Scale (GOS) or generic HRQoL instruments because a TBI-specific HRQoL instrument was not available [32, 48]. These methods however lacked the perspective of subjective well-being and were considered to be less sensitive [46]. To overcome these limitations, the Quality Of Life after Brain Injury questionnaire (QOLIBRI) was developed [46]. This TBI-specific HRQoL measure covers six dimensions typically affected after TBI and provides more precise information on quality of life [46]. It has been validated in multiple study settings but has not been used frequently to measure outcome after t-ASDH in clinical studies [45]. Therefore, the TBI-specific HRQoL was investigated in addition to functional outcome (GOS) after the surgical treatment of patients with a t-ASDH.

Furthermore, we analyzed the in-hospital costs associated with both conservative and different surgical treatments in patients with a diagnosed t-ASDH. Costs for the treatment of TBI are high and annually increasing. In the US, for example, the national hospital costs for all subdural hematomas were estimated to be $US1.6 billion in 2007, a 60% increase compared to 1998 [10]. There is an increasing pressure from governments, insurance companies, and healthcare providers to control healthcare costs [30]. The demand for high-quality evidence regarding the cost-effectiveness of treatments is also seen in TBI, where it lacks and where expensive life-saving surgical treatments can also result in a poor HRQoL [2, 22].

Because patient outcome and in-hospital costs of patients with a t-ASDH are of great individual and societal importance, the aim of this study is threefold: (1) assess functional outcome and TBI-specific HRQoL, (2) calculate the in-hospital costs, and (3) serve as a basis for future research that focusses on the cost-effectiveness of surgical treatment of patients with t-ASDH.

## Methods and materials

### Study setting

This retrospective cohort study was conducted at the neurosurgical departments of two collaborating level I trauma centers in The Netherlands (Leiden University Medical Center, Leiden, and Haaglanden Medical Center, The Hague). The study reports in-hospital costs and long-term HRQoL follow-up data of patients that are part of a cohort partly used in a separate study by the same investigators [44]. The research ethics committees of Southwest Holland and Leiden University Medical Center provided ethical approval (study number P12.196).

### Patients

All consecutive patients with TBI (2008–2012) treated by the department of neurosurgery were identified by screening the hospital registration system. In addition, the national trauma registry was checked for potential missed inclusions. Inclusion criteria were (1) closed head injury due to a traumatic event, (2) direct presentation to the emergency department of a referring or study hospital following trauma, (3) a hyperdense, crescent-shaped lesion on CT, indicative of an ASDH, and (4) age ≥ 16 years. To pursue a homogenous patient cohort, patients were excluded in case of non-survivable extracranial injuries, a non-traumatic ASDH, when the ASDH was accompanied by concomitant intracranial lesions (i.e., intracerebral hematoma or epidural hematoma) requiring immediate surgical management and when the ASDH was secondary to an earlier procedure or penetrating brain injury. Eligibility, the QOLIBRI questionnaire was assessed based on exclusion criteria: GOS ≤ 3, inability to provide informed consent and inability to understand, cooperate, and answer QOLIBRI questions. TBI severity was defined according to the commonly used Glasgow Coma Score scale (GCS) categories (GCS13–15, mild; GCS 9–12, moderate; GCS 3–8, severe) [39]. In addition, a subgroup of patients with a very severe TBI (vs-TBI), represented by a GCS of 3–5, was analyzed. The first GCS score documented at the emergency room (ER) was used and in case of intubation and/or sedation, the last score before intubation and/or sedation was used.

### Clinical and follow-up data

Data was collected independently by two authors in a predefined database using electronic or paper patient records. It encompassed demographics, patient- and trauma-specific information, and pre- and in-hospital parameters including medical/surgical interventions and length of stay. Non-ICU admission included admission on the ward and medium care. Focal neurologic symptoms included paresis, aphasia, or cranial nerve deficit. Pupils were defined as abnormal when at least one pupil was unresponsive to light upon arrival in the emergency room. CT characteristics were assessed from the first CT scan. Outcome data included in-hospital mortality and Glasgow Outcome Score (GOS) dichotomized in favorable (GOS 4–5) and unfavorable (GOS 1–3) outcome obtained from discharge or outpatient clinic letters 3–9 months after trauma [48]. To determine the TBI-specific HRQoL, we used the postal Quality of Life after Brain Injury (QOLIBRI) questionnaire. After receiving ethical approval to approach patients, we obtained informed consent and asked patients to complete and return the questionnaire 2 to 6 years after trauma. Mortality at this time point was also noted. The QOLIBRI is a comprehensive 37-item questionnaire investigating six dimensions that are typically affected after TBI [46]. Patients rate their (dis)satisfaction (1–5 scale) on six subscales representing the dimensions: cognition, self, daily life and autonomy, social relationships, emotions, and physical problems. Scores are transformed to total scores ranging from 0 (worst possible quality of life) to 100 (best possible quality of life) [46]. A score lower than 60 is believed to represent a low or impaired HRQoL [49]. In case patients did not return the questionnaire, the investigators attempted a telephone interview, or family members were asked to assist in completing the forms. In addition, the reason for not returning (e.g., death, persistent unresponsive state) the questionnaire was collected at this time point.

### Cost data

Cost data analysis was performed from a healthcare provider perspective and focused on in-hospital healthcare costs. The Dutch National Health Care Institute guidelines for healthcare cost calculation were followed [14]. First, data on healthcare consumption were collected from electronic patient records and recorded in a predefined cost assessment database. Units were counted in five main categories: (1) admission, including length of stay (LOS) in (non-)ICU with consultations, (2) surgical interventions, (3) imaging, (4) laboratory, including blood products, and (5) others, including transportation and outpatient visits. Since this study focused on in-hospital acute healthcare costs, only post-discharge costs associated with re-admissions and outpatient clinic visits related to the initial trauma were included. Second, as hospital-specific cost prices were not available for external research purposes, units were valued by using external sources in accordance with the guidelines [14]. Some units were valued using the reference prices from the guideline, being cost prices based on large patient cohorts [14]. The use of these prices is recommended for costs research and preferred for cost outcome interpretation and generalization because prices are non-site-specific [14, 37]. Units that were not available in the guidelines were valued using the maximum amount per unit that healthcare providers are allowed to charge according to the The Netherlands Healthcare Authority (NZa), an autonomous administrative authority falling under the Dutch Ministry of Health, Welfare and Sport [29]. The remaining units were valued by using their average national price based on declared fees including hospital costs and physicians’ fees [28]. A detailed overview of all used unit costs and corresponding sources can be found in Supplement 1.

Third, we corrected all unit costs expressed in different base years to 2012 EURO using the national general consumer price index (CBS). This year was chosen because it was the last year of patient inclusion. And finally, to calculate in-hospital costs, all counted units were multiplied with its corresponding price and rounded to the nearest ten euros. No discounting of costs was deemed necessary. In January 2012, one euro equaled $1.28.

### Statistical analysis

Baseline data were presented as absolute numbers and percentages. Continues variables, like costs and LOS, were presented as mean ± standard deviation unless stated otherwise. Subgroups were made based on age, TBI severity, pupillary abnormalities, surgical intervention, and outcome. Comparison between groups was done by using an independent *t* test. A *p* value of < 0.05 was considered statistically significant. All analyses were performed using IBM’s statistical package for social sciences version 23 (SPSS). Figures were designed with GraphPad Prism version 7.02.

## Results

Out of 294 initially identified TBI patients, 140 patients did not have a t-ASDH, six had penetrating injuries, nine required surgery for concomitant intracranial lesions, and 31 patients were excluded following the other exclusion criteria. Ultimately, 108 patients were included in this study. The final study cohort included 57 males (52.8%) and had a mean age of 65 years (range 18–91) (Table 1). Most ASDH patients (*N* = 49) sustained a severe TBI (s-TBI) followed by mild (*N* = 40) and moderate TBI (*N* = 19). Of patients with s-TBI, 22 were classified as having sustained a vs-TBI. A quarter of all patients had at least one non-reactive pupil (*N* = 27) and 38.9% had focal neurologic symptoms. A concomitant intracranial hematoma that did not require surgical intervention was present in 44.4% of patients and 11.1% had clinically relevant extracranial injuries. Neurosurgical intervention was performed in 90 patients (60 craniotomies, 29 decompressive craniectomies, and one burr hole) and an ICP monitoring device was placed in 40 patients. Most of the conservatively treated patients (*N* = 18) were classified as mild TBI (83%).

**Table 1:** Patient cohort information

Number of patients	108
Age (years)	65 ± 17.3
Male	57 (52.8)
Trauma mechanism	
Fall	58 (53.7)
Assault	5 (4.6)
Motor vehicle accident	12 (11.1)
Fall from bike	12 (11.1)
Other	21 (19.4)
TBI severity	
Very severe (GCS3–5)	22 (20.4)
Severe (GCS3–8)	49 (45.4)
Moderate (GCS9–12)	19 (17.6)
Mild (GCS13–15)	40 (37.0)
Clinical parameters	
GCS score	9.63 ± 4.3
Pupil abnormality^*^	27 (26.7)
Focal neurologic symptoms	42 (38.9)
Major extracranial injury	12 (11.1)
CT parameters	
Thickness (mm)	13.6 ± 6.1
Midline shift (mm)	11.4 ± 6.6
Concomitant lesion	48 (44.4)
Basal cisterns compressed	39 (36.1)
Treatment	
Conservative	18 (16.7)
Emergent surgical intervention:	90 (83.3)
Craniotomy	− 60 (55.6)
Decompressive craniectomy (DC)	− 29 (26.9)
ICP monitoring	− 40 (37.0)
In-hospital mortality	41 (37.9)
Functional outcome	
GOS1–3 (unfavorable)	56 (51.9)
GOS4–5 (favorable)	50 (46.3)
Missing GOS	2 (1.9)
QOLIBRI response	
FU time, months	46 ± 16
Yes	25 (23.1)
No (died; too disabled)	53 (48; 5)
No, other	30 (27.8)

Table 1 provides general information about the patient cohort. Legend: *N* (%) or mean **±** SD, unless stated otherwise

*SD*, standard deviation; *GCS*, Glasgow Coma Score; *CT*, computed tomography; *DC*, decompressive craniectomy; *ICP*, intracranial pressure; *GOS*, Glasgow Outcome Score; *QOLIBRI*, quality of life after brain injury; *FU*, follow-up

^*^At least one pupil unresponsive to light upon arrival in the emergency room (missing for seven patients)

### Patient outcome

**Fig. 1:**
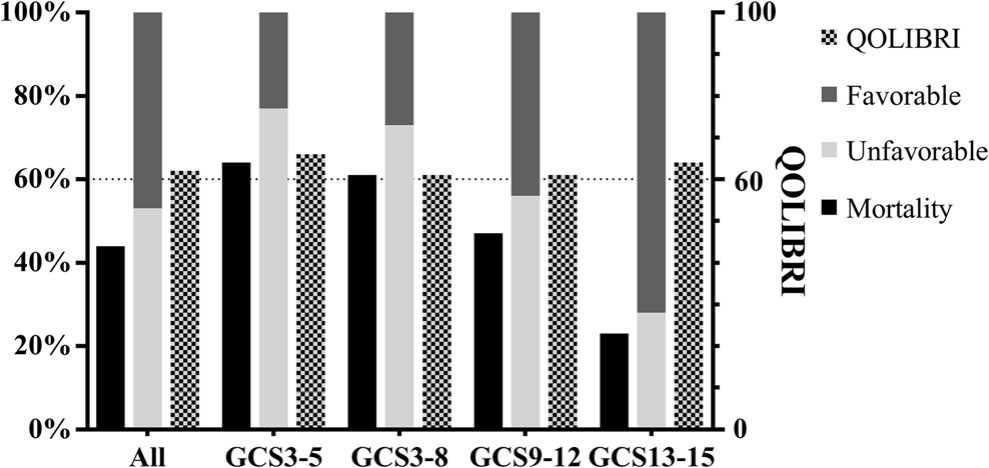
Functional outcome (favorable GOS 4–5, unfavorable GOS 1–3) and TBI-specific health-related quality of life (QOLIBRI) for all patients and for severity subgroups

In-hospital mortality was 38% and mortality increased to 44% during follow-up (mean 37 ± 17 months). Mortality ranged from 23% for initial mild-TBI to 64% for patients with vs-TBI (Table 2). Favorable outcome (GOS 4–5) was seen in 47% of all patients, 72% of patients with mild-TBI, and in 23% of patients with vs-TBI (Fig. 1). High rates of unfavorable outcome (GOS 1–3) were seen in patients with a GCS of 3 (90%), ICP monitoring (75%), decompressive craniectomy (72%), pupillary abnormalities (70%), and age < 65 (63%).

**Table 2:** Patient outcome

Patient category	*N*	*N* (%) death ^*^*^	*N* (%) GOS1–3	*N* (%) returned QOLIBRI^#^	QOLIBRI score	QOLIBRI follow-up (months)
All patients	108	48 (44)	56 (53)	25 (23)	62.8 ± 23.5	37 ± 17
Age ≥ 65	65	21 (32)	29 (45)	16 (25)	66.8 ± 22.1	38 ± 18
Age < 65	43	19 (44)	27 (63)	9 (21)	55.7 ± 25.6	35 ± 16
GCS 3	10	7 (70)	9 (90)	0	N/A	N/A
GCS 3–5	22	14 (64)	17 (77)	2 (9)	66.0 ± 7.07	13 ± 2
GCS 3–8	49	30 (61)	35 (71)	7 (14)	61.4 ± 24.8	34 ± 19
GCS 9–12	19	9 (47)	10 (53)	4 (21)	61.0 ± 25.5	50 ± 21
GCS 13–15	40	9 (23)	11 (28)	14 (35)	64.0 ± 24.1	35 ± 14
Pupillary abnormality	27	15 (56)	19 (70)	5 (19)	49.8 ± 19.4	47 ± 23
No abnormalities*	74	29 (39)	32 (43)	18 (24)	64.5 ± 24.6	32 ± 13
Emergency surgery						
No	18	3 (17)	3 (17)	4 (22)	56.3 ± 28.6	33 ± 15
Craniotomy	60	26 (43)	32 (53)	15 (25)	68.4 ± 21.0	36 ± 17
Decompressive craniectomy	29	18 (62)	21 (72)	6 (21)	53.2 ± 26.3	42 ± 21
ICP monitoring	40	20 (50)	30 (75)	9 (23)	55.1 ± 20.4	36 ± 24
No ICP monitoring	68	28 (41)	26 (38)	16 (24)	67.1 ± 24.7	37 ± 13
Outcome (GOS)						
Favorable	50	4 (8)	N/A	23 (46)	63.9 ± 23.3	37 ± 17
Unfavorable	56	42 (75)	56 (100)	2 (4)		37 ± 25
Missing	2				50.5 ± 2.1	

Table 2 provides an overview of mortality, functional outcome and health related quality of life per subgroup. Legend: results presented as number (row percentage) and mean ± SD

^#^The response rate is reported as percentage of survivors from the specific category

*Pupillary abnormality information was missing for seven patients

^Mortality at time of QOLIBRI follow-up

*LOS*, length of stay; *GCS*, Glasgow Coma Score; *ICP*, intracranial pressure; *QOLIBRI*, quality of life after brain injury; *M*, months; *N/A*, not applicable

Twenty-five patients (42% of survivors) returned a completed QOLIBRI questionnaire. Return percentages were lower for patients with higher initial severity scores (9% for vs-TBI and 35% for mild TBI) and lower for patients with worse functional outcome (4% for GOS 1–3 vs. 46% for GOS 4–5). Mean QOLIBRI scores however were rather similar between TBI severity groups (61 ± 25 for s-TBI and 64 ± 24 for mild TBI). Patients with post-trauma pupillary abnormalities (49.8), ICP monitoring (55.1), and patients with unfavorable outcome (GOS 1–3) (50.5) showed mean QOLIBRI scores suggesting an impaired HRQoL. Patients receiving a craniotomy showed better scores (68.4) than patients receiving a decompressive craniectomy (53.2).

### Healthcare consumption

Patients with vs-TBI had a significantly longer ICU LOS than patients with mild TBI (6 vs. 2 days, *P* < 0.001) (Table 3). Mean LOS for non-ICU admissions was longest for patients with moderate TBI (16 days), followed by 12 and 9 days for patients with vs-TBI and mild TBI. All vs-TBI and 98% of s-TBI patients received cranial surgery, compared to 89.5% of moderate and 62.5% of mild TBI patients. ICP monitoring was most frequently used in patients with vs-TBI and s-TBI (63.6% and 57.1%), but also in 12.5% of patients with mild TBI. ICP monitoring was associated with significantly longer ICU and non-ICU LOS compared to non-ICP-monitoring.

**Table 3:** Length of stay and in-hospital costs

Patient category	N	ICU LOS	Non-ICU LOS	Total costs (€)	Admission costs	Surgery costs
All patients	108	4 ± 4	11 ± 14	24,980 ± 17,060	14,980 ± 14,000	6890 ± 4270
Age ≥ 65	65	3 ± 3	10 ± 12	20,820 ± 13,480	11,750 ± 10,670	6150 ± 4040
Age < 65	43	6 ± 5	12 ± 16	31,260 ± 19,930	19,850 ± 16,890	8020 ± 4410
GCS 3	10	3 ± 3	11 ± 19	24,690 ± 18,020	13,720 ± 16,310	7940 ± 2340
GCS 3–5	22	6 ± 4	12 ± 17	30,230 ± 16,370	19,110 ± 14,910	7710 ± 1750
GCS 3–8	49	6 ± 5	11 ± 14	29,660 ± 17,870	18,780 ± 15,890	7520 ± 2200
GCS 9–12	19	3 ± 3	16 ± 20	27,650 ± 15,780	15,120 ± 12,600	9230 ± 5470
GCS 13–15	40	2 ± 4	9 ± 8	17,980 ± 14,460	10,250 ± 10,610	5010 ± 4840
Pupillary abnormality	27	7 ± 5	13 ± 14	33,430 ± 18,330	22,480 ± 16,850	7510 ± 1600
No abnormalities	74	3 ± 4	11 ± 14	22,220 ± 16,110	12,590 ± 12,120	6690 ± 4940
Emergency surgery	90	5 ± 5	12 ± 15	28,670 ± 16,230	17,120 ± 14,290	8270 ± 3220
No	18	1 ± 2	4 ± 5	6520 ± 4320	4240 ± 4160	0
Craniotomy	60	4 ± 4	12 ± 14	26,400 ± 14,680	16,040 ± 12,790	7310 ± 3060
DC	29	6 ± 5	11 ± 16	33,140 ± 19,070	19,950 ± 16,980	9550 ± 3790
ICP monitoring	40	7 ± 5	15 ± 16	36,580 ± 16,650	23,420 ± 15,260	9340 ± 3730
No ICP monitoring	68	2 ± 3	9 ± 12	18,150 ± 13,250	10,010 ± 10,480	5460 ± 3920
Outcome						
Favorable*	50	3 ± 4	11 ± 10	20,430 ± 16,540	12,320 ± 13,170	5270 ± 3910
Unfavorable	56	5 ± 5	11 ± 16	29,230 ± 16,850	17,650 ± 14,490	8230 ± 4100
Dead at discharge	41	5 ± 4	6 ± 10	25,340 ± 12,450	13,890 ± 10,070	8180 ± 3770

Table 3 provides an overview of length of stay and in-hospital costs per subgroup. In-hospital costs are divided between costs related to admission and surgical intervention. Mean ± SD; all costs in € and LOS in days

*GOS outcomes not available for two patients

*N*, number; *LOS*, length of stay; *GCS*, Glasgow Coma Score; *ICU*, Intensive Care Unit; *DC*, decompressive craniectomy; *ICP*, intracranial pressure

### Healthcare costs

**Fig. 2:**
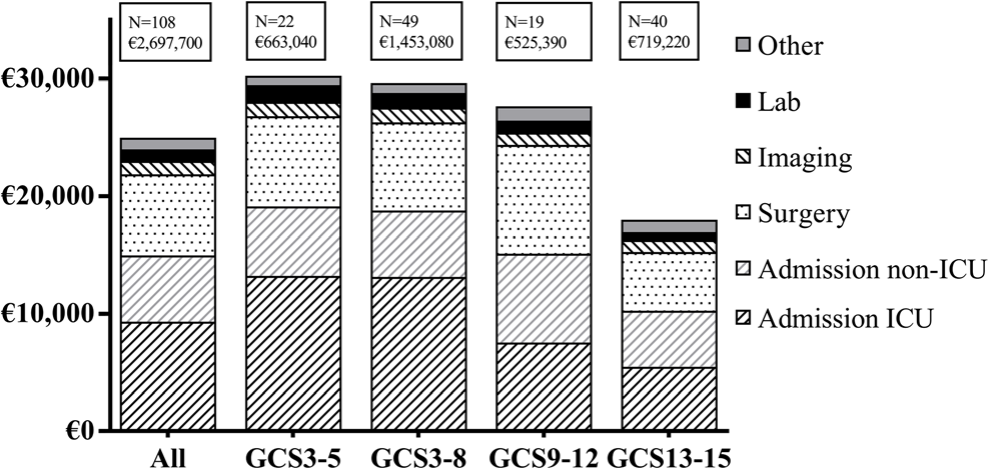
Mean and total in-hospital costs for all patients and for severity subgroups. Also, a distinction has been made between investigated cost categories to show their share to the total in-hospital costs

Mean in-hospital costs were €24,980 per patient and primarily the result of costs related to admission (€14,980) and surgical intervention (€6890). Mean in-hospital costs were significantly higher for vs-TBI (€30,230), s-TBI (€29,660), and moderate TBI (€27,650) subgroups compared to the mild TBI (€17,980) subgroup (*P* < 0.05) (Table 3). For these severity subgroups, mean costs specifically related to ICU admission were €13,230, €13,150, €7550, and €5460 respectively (Fig. 2). Patients’ healthcare utilization was more expensive after surgical intervention than conservative treatment (€28,670 vs. €6520). Patients with a decompressive craniectomy showed the highest cost specifically related to surgery. Patients with additional ICP monitoring (€36,580) showed highest total costs, of which 64% was related to admission. A lower initial GCS and pupillary abnormalities show an increase in patient LOS and in-hospital costs, except for patients with a GCS of 3. Other characteristics associated with significantly increased total costs were: age < 65, a concomitant intracranial hematoma that did not require surgical intervention, presence of pupillary abnormalities, and unfavorable outcome.

Five patients (23%) from the vs-TBI subgroup achieved favorable outcome (GOS4–5) at mean in-hospital costs of €132,610 per patient. Mean costs for patients achieving favorable outcome were €103,790 for s-TBI patients (*N* = 14; 29%), €58,150 for moderate TBI patients (*N* = 9; 47%), and €24,800 per mild-TBI patient (*N* = 29; 72%). Mean in-hospital costs were highest (€246,920) for one patient from the GCS = 3 subgroup (*N* = 10) that reached favorable outcome.

## Discussion

“Favorable” outcome with a good HRQoL was achieved in an important quarter proportion of the seemingly most severely injured patients. This retrospective cohort study, however, also shows high rates of mortality and so-called unfavorable outcome in patients with a t-ASDH and relatively high healthcare consumption and in-hospital costs. These costs increased with higher injury severity scores and in patients with surgical intervention. The majority of costs were related to (ICU) admission and surgical intervention. According to the investigators, this study shows a trend that surgical treatment of t-ASDH can realize favorable outcome in s-TBI at for society acceptable in-hospital costs.

### Patient outcome

Accurate comparison of the reported patient outcome results with literature is challenging because outcome in TBI is highly variable and dependent on patient characteristics, circumstances, social context, and treatment [7, 9, 13, 21, 31]. Nonetheless, the important result that even the most severely injured TBI patients can, although a small number, achieve favorable outcome (GOS) and good quality of life (QOLIBRI) is supported by recent literature [36, 42].

Our QOLIBRI results are not applicable to study patients with a cognitive dysfunction and/or impaired self-awareness that is too severe to complete the questionnaire. The unmeasured HRQoL of these patients might have negatively influenced the reported HRQoL per TBI severity group. The applicability of the QOLIBRI for all patients with TBI remains unclear since it has only been validated in patients without substantial post-traumatic cognitive restraints [45]. Proxy completion is impossible for many QOLIBRI items and misses the essence of measuring the “self-perceived” HRQoL. It also remains unclear whether the cut-off point of 60 is satisfying for quantifying a good HRQoL [49]. Therefore, validity should be confirmed for patients with TBI-associated persisting cognitive restraints or suitable new (HRQoL) measurement options need to be developed.

In contrast to earlier published reports on t-ASDH, the mean cohort age of 65 years was relatively high, but in accordance with changing TBI epidemiology [25]. Also, a large number of patients had an initial low GCS and/or pupillary abnormalities. These three factors are known to negatively influence outcome and sometimes these patients are even considered unsalvageable [9, 13, 42]. Nevertheless, neurosurgical intervention was performed in up to 98% of patients with s-TBI. This percentage is high compared to other studies, but seems rational, since neurosurgical evacuation of the hematoma and/or DC can be lifesaving and prevent secondary injury by decreasing ICP [9, 20, 21, 23]. The high percentage can also be explained by the specific selection of patients with a t-ASDH where neurosurgical consultation was considered necessary, suggesting a higher vulnerability. Although the present study did not evaluate treatment effectiveness, a separate analysis by the authors seemed to support the more aggressive approach [44]. Even so, superiority between hematoma evacuation or DC remains unknown and no clinical trial has proven primary DC to be effective in improving patient outcome [17, 31]. Surgical intervention is even controversial because patients may survive with “unacceptable” severe disabilities with an accompanying high burden on proxies and society [16]. This is fundamental in neurosurgical treatment decision-making and as a result, a “surgical” treatment strategy as seen in this study, which follows the guidelines, is not a standard day-to-day care in all hospitals [9, 20, 43, 44].

Instead, treatment-limiting decisions in s-TBI are common in some countries and often made within the first 2 days after trauma [35, 50]. Limiting treatment offers no serious chance of recovery and regularly results in quick death [35, 50]. We acknowledge that these decisions are sometimes inevitable and could be in a patients’ best interest when there is no realistic chance to achieve a “favorable” outcome. But what can be considered a favorable or an unfavorable outcome after s-TBI and vs-TBI?

Therefore, according to the investigators, it would be catastrophic to limit or withhold treatment in patients that could have still benefitted from it. Physicians should be careful in making early treatment limiting decisions when there is still uncertainty, because uncertainty implies a possibility for favorable outcome. Unfortunately, uncertainty in predicting who will benefit from what treatment is very common. There is substantial variation in the perception of neurologic prognosis among physicians and high treatment variation [7, 41, 43]. In line with some literature, we believe that treatment-limiting decisions in the early phase cannot be justified because prognostication is not yet accurate enough [12]. In a later stage, when clinical and neurological improvements remain absent, further treatment might be considered futile with more certainty. Then, treatment-limiting decisions should be discussed with all involved healthcare professionals and proxies.

### Healthcare consumption and in-hospital costs

The costs related to admission and surgical intervention cost categories appeared to be the most important contributors to the reported in-hospital costs. In literature, costs related to ICU admission were also high and in-hospital costs also increased with higher injury and TBI severity (defined by GCS), ICP monitoring, and surgical intervention [3, 8, 27, 34, 38]. The surprisingly lower LOS and in-hospital costs for elderly patients in this study could be explained by the fact that only 33.8% of elderly patients was classified as severe, compared to 62.8% of patients younger than 65.

Overall, the reported healthcare consumption and in-hospital costs seem to be quite similar to literature [18, 34, 38]. However, comparison was difficult due to substantial methodological variation and often inadequate methodology of available TBI cost studies [2, 22]. The detailed calculation of healthcare consumption and in-hospital costs is an important strength of this study. The electronic patient file setup reduced the risk to a minimum that unregistered activities contributed to an underestimation of in-hospital resource utilization. Still, the numbers in this study are an enormous underestimation of the total healthcare consumption and total costs associated with t-ASDH and TBI because the majority of costs are indirect and arise after hospital discharge [11, 34, 40]. Also, interpretation and generalization of the results should be done carefully since included patients represent a specific selection of patients with a t-ASDH with a suspected higher vulnerability, where patients with a concomitant hematoma requiring surgical intervention were excluded. Also, the inevitable presence of coexisting injuries causes that results are not solely attributable to TBI.

Despite these remarks, the reported costs give rise to the question whether or not the in-hospital costs may be justified by the achieved outcome. The mean in-hospital costs per patient appear to be acceptable for all TBI severity groups. However, when adding up the in-hospital costs that are made to have one patient achieve a favorable outcome, especially for the most severely injured patients appear to be expensive. Unfortunately, true cost-effectiveness could not be established in this study, and because there is no consensus in literature, additional research is needed to establish cost-effectiveness and justification of expenses in TBI care [1, 15, 26, 47].

### Future perspective

Future research should establish long-term outcome of ASDH patients after different treatment strategies. A high-quality cost-effectiveness research should incorporate a long-term follow-up and should use accurate resource utilization and cost price information [19, 33]. Future research should also explore the societal impact of t-ASDH, including productivity loss of both patients and proxies. Investigators should aim at comparability and generalizability by using common data points and guideline recommendations [24]. Ultimately, researchers should explore what health states and associated costs can be considered “acceptable” to patients, proxies, and society.

## Conclusions

Although outcome was often “unfavorable,” several of the most severely injured patients, often even considered unsalvageable, achieved favorable outcome on both GOS and QOLIBRI. Associated hospital costs were relatively high, especially for the most severely injured patients, but may be justified considering the realized favorable outcome in part of these patients. Patients should not prematurely be considered unsalvageable and adequate (surgical) therapy should not be withheld in the acute phase. More research is necessary to establish the cost-effectiveness of treatment strategies for patients with a t-ASDH.

## Electronic supplementary material

ESM 1 (DOCX 21 kb)

## Abbreviations

CBS: Central bureau of statistics
CT: Computed tomography
DC: Decompressive craniectomy
ER: Emergency room
GCS: Glasgow Coma Scale
GOS: Glasgow Outcome Score
HRQoL: Health-related quality of life
ICP: Intracranial pressure
ICU: Intensive care unit
LOS: Length of stay
NZa: Netherlands Healthcare authority
QOLIBRI: Quality of Life after Brain Injury
t-ASDH: Traumatic acute subdural hematoma
TBI: Traumatic brain injury
TLD: Treatment limiting decisions
US: United States
Vs-TBI: Very severe traumatic brain injury

## References

[1] Alali AS, Naimark DM, Wilson JR, Fowler RA, Scales DC, Golan E, Mainprize TG, Ray JG, Nathens AB (2014). Economic evaluation of decompressive craniectomy versus barbiturate coma for refractory intracranial hypertension following traumatic brain injury. Crit Care Med.

[2] Alali AS, Burton K, Fowler RA, Naimark DM, Scales DC, Mainprize TG, Nathens AB (2015). Economic evaluations in the diagnosis and management of traumatic brain injury: a systematic review and analysis of quality. Value Health.

[3] Albrecht JS, Slejko JF, Stein DM, Smith GS (2017). Treatment charges for traumatic brain injury among older adults at a trauma center. J Head Trauma Rehabil.

[4] Bullock MR, Chesnut R, Ghajar J, Gordon D, Hartl R, Newell DW, Servadei F, Walters BC, Wilberger JE (2006). Surgical management of acute subdural hematomas. Neurosurgery.

[5] Carney N, Totten AM, O'Reilly C, Ullman JS, Hawryluk GW, Bell MJ, Bratton S, Chesnut R, Harris OA, Kissoon N, Rubiano AM, Shutter L, Tasker RC, Vavilala MS, Wilberger J, Wright DW, Ghajar J (2017). Guidelines for the management of severe traumatic brain injury, fourth edition. Neurosurgery.

[6] Cnossen MC, Scholten AC, Lingsma HF, Synnot A, Tavender E, Gantner D, Lecky F, Steyerberg EW, Polinder S (2016) Adherence to guidelines in adult patients with traumatic brain injury: a living systematic review. J Neurotrauma. 10.1089/neu.2015.4121 10.1089/neu.2015.4121 PMC8054518 26431625

[7] Cnossen MC, Polinder S, Andriessen TM, van der Naalt J, Haitsma I, Horn J, Franschman G, Vos PE, Steyerberg EW, Lingsma HF (2017). Causes and consequences of treatment variation in moderate and severe traumatic brain injury: a multicenter study. Crit Care Med.

[8] Fountain DM, Kolias AG, Laing RJ, Hutchinson PJ (2016). The financial outcome of traumatic brain injury: a single centre study. Br J Neurosurg.

[9] Fountain DM, Kolias AG, Lecky FE, Bouamra O, Lawrence T, Adams H, Bond SJ, Hutchinson PJ (2017). Survival trends after surgery for acute subdural hematoma in adults over a 20-year period. Ann Surg.

[10] Frontera JA, Egorova N, Moskowitz AJ (2011). National trend in prevalence, cost, and discharge disposition after subdural hematoma from 1998-2007. Crit Care Med.

[11] Garcia-Altes A, Perez K, Novoa A, Suelves JM, Bernabeu M, Vidal J, Arrufat V, Santamarina-Rubio E, Ferrando J, Cogollos M, Cantera CM, Luque JC (2012). Spinal cord injury and traumatic brain injury: a cost-of-illness study. Neuroepidemiology.

[12] Geurts M, Macleod MR, van Thiel GJ, van Gijn J, Kappelle LJ, van der Worp HB (2014). End-of-life decisions in patients with severe acute brain injury. Lancet Neurol.

[13] Greene NH, Kernic MA, Vavilala MS, Rivara FP (2018). Variation in adult traumatic brain injury outcomes in the United States. J Head Trauma Rehabil.

[14] Hakkaart-van Roijen L, van der Linden N, Bouwmans C, Kanters T, Tan S (2015) Kostenhandleiding: methodologie van kostenonderzoek en referentieprijzen voor economische evaluaties in de gezondheidszorg. Zorginstituut Nederland Geactualiseerde versie 2015 https://www.zorginstituutnederland.nl/binaries/zinl/documenten/publicatie/2016/02/29/richtlijn-voor-het-uitvoeren-van-economische-evaluaties-in-de-gezondheidszorg/Richtlijn+voor+het+uitvoeren+van+economische+evaluaties+in+de+gezondheidszorg+%28verdiepingsmodules%29.pdf. Accessed March 29 2018

[15] Ho KM, Honeybul S, Lind CR, Gillett GR, Litton E (2011). Cost-effectiveness of decompressive craniectomy as a lifesaving rescue procedure for patients with severe traumatic brain injury. J Trauma.

[16] Honeybul S, Gillett G, Ho K, Lind C (2012). Ethical considerations for performing decompressive craniectomy as a life-saving intervention for severe traumatic brain injury. J Med Ethics.

[17] Hutchinson PJ, Kolias AG, Timofeev IS, Corteen EA, Czosnyka M, Timothy J, Anderson I, Bulters DO, Belli A, Eynon CA, Wadley J, Mendelow AD, Mitchell PM, Wilson M, Critchley G, Sahuquillo J, Unterberg A, Servadei F, Teasdale GM, Pickard JD, Menon DK, Murray GD, Kirkpatrick PJ (2016). Trial of decompressive craniectomy for traumatic intracranial hypertension. N Engl J Med.

[18] Kalanithi P, Schubert RD, Lad SP, Harris OA, Boakye M (2011). Hospital costs, incidence, and inhospital mortality rates of traumatic subdural hematoma in the United States. J Neurosurg.

[19] Keel G, Savage C, Rafiq M, Mazzocato P (2017). Time-driven activity-based costing in health care: a systematic review of the literature. Health Policy.

[20] Kwon H, Choi KS, Yi HJ, Chun HJ, Lee YJ, Kim DW (2017). Risk factors of delayed surgical intervention after conservatively treated acute traumatic subdural hematoma. J Korean Neurosurg Soc.

[21] Leitgeb J, Mauritz W, Brazinova A, Janciak I, Majdan M, Wilbacher I, Rusnak M (2012). Outcome after severe brain trauma due to acute subdural hematoma. J Neurosurg.

[22] Lu J, Roe C, Aas E, Lapane KL, Niemeier J, Arango-Lasprilla JC, Andelic N (2013). Traumatic brain injury: methodological approaches to estimate health and economic outcomes. J Neurotrauma.

[23] Maas AI, Stocchetti N, Bullock R (2008). Moderate and severe traumatic brain injury in adults. Lancet Neurol.

[24] Maas AI, Harrison-Felix CL, Menon D, Adelson PD, Balkin T, Bullock R, Engel DC, Gordon W, Langlois Orman JL, Lew HL, Robertson C, Temkin N, Valadka A, Verfaellie M, Wainwright M, Wright DW, Schwab K (2010). Common data elements for traumatic brain injury: recommendations from the interagency working group on demographics and clinical assessment. Arch Phys Med Rehabil.

[25] Maas AIR, Menon DK, Adelson PD, Andelic N, Bell MJ, Belli A, Bragge P, Brazinova A, Buki A, Chesnut RM, Citerio G, Coburn M, Cooper DJ, Crowder AT, Czeiter E, Czosnyka M, Diaz-Arrastia R, Dreier JP, Duhaime AC, Ercole A, van Essen TA, Feigin VL, Gao G, Giacino J, Gonzalez-Lara LE, Gruen RL, Gupta D, Hartings JA, Hill S, Jiang JY, Ketharanathan N, Kompanje EJO, Lanyon L, Laureys S, Lecky F, Levin H, Lingsma HF, Maegele M, Majdan M, Manley G, Marsteller J, Mascia L, McFadyen C, Mondello S, Newcombe V, Palotie A, Parizel PM, Peul W, Piercy J, Polinder S, Puybasset L, Rasmussen TE, Rossaint R, Smielewski P, Soderberg J, Stanworth SJ, Stein MB, von Steinbuchel N, Stewart W, Steyerberg EW, Stocchetti N, Synnot A, Te Ao B, Tenovuo O, Theadom A, Tibboel D, Videtta W, Wang KKW, Williams WH, Wilson L, Yaffe K (2017). Traumatic brain injury: integrated approaches to improve prevention, clinical care, and research. Lancet Neurol.

[26] Malmivaara K, Kivisaari R, Hernesniemi J, Siironen J (2011). Cost-effectiveness of decompressive craniectomy in traumatic brain injuries. Eur J Neurol.

[27] Marin JR, Weaver MD, Mannix RC (2017). Burden of USA hospital charges for traumatic brain injury. Brain Inj.

[28] Nederlandse Zorgautoriteit. Open data van de Nederlandse Zorgautoriteit. http://www.opendisdata.nl. Accessed March 29 2018

[29] Nederlandse Zorgautoriteit Tarieventabel DBC-zorgproducten en overige producten - per 1 januari 2012 (PUC_12710_22). https://puc.overheid.nl/nza/doc/PUC_12710_22/1/. Accessed March 29 2018

[30] Parlementary letter, Ministry of Health (2016). https://www.rijksoverheid.nl/documenten/kamerstukken/2016/01/28/kamerbrief-over-beeindiging-sluis-nivolumab-per-1-maart-2016. Accessed July 30 2018

[31] Phan K, Moore JM, Griessenauer C, Dmytriw AA, Scherman DB, Sheik-Ali S, Adeeb N, Ogilvy CS, Thomas A, Rosenfeld JV (2017). Craniotomy versus decompressive craniectomy for acute subdural hematoma: systematic review and meta-analysis. World Neurosurg.

[32] Polinder S, Haagsma JA, van Klaveren D, Steyerberg EW, van Beeck EF (2015). Health-related quality of life after TBI: a systematic review of study design, instruments, measurement properties, and outcome. Popul Health Metrics.

[33] Porter ME, Lee TH (2013) The strategy that will fix health care. Harv Bus Rev https://hbr.org/2013/10/the-strategy-that-will-fix-health-care. Accessed March 30 2018

[34] Raj R, Bendel S, Reinikainen M, Hoppu S, Laitio R, Ala-Kokko T, Curtze S, Skrifvars MB (2018). Costs, outcome and cost-effectiveness of neurocritical care: a multi-center observational study. Crit Care.

[35] Robertsen A, Førde R, Skaga NO, Helseth E (2017). Treatment-limiting decisions in patients with severe traumatic brain injury in a Norwegian regional trauma center. Scand J Trauma Resusc Emerg Med.

[36] Soberg HL, Roe C, Anke A, Arango-Lasprilla JC, Skandsen T, Sveen U, von Steinbuchel N, Andelic N (2013). Health-related quality of life 12 months after severe traumatic brain injury: a prospective nationwide cohort study. J Rehabil Med.

[37] Tan SS, Bakker J, Hoogendoorn ME, Kapila A, Martin J, Pezzi A, Pittoni G, Spronk PE, Welte R, Hakkaart-van Roijen L (2012). Direct cost analysis of intensive care unit stay in four European countries: applying a standardized costing methodology. Value Health.

[38] Tardif PA, Moore L, Boutin A, Dufresne P, Omar M, Bourgeois G, Bonaventure PL, Kuimi BL, Turgeon AF (2017). Hospital length of stay following admission for traumatic brain injury in a Canadian integrated trauma system: a retrospective multicenter cohort study. Injury.

[39] Teasdale G, Jennett B (1974). Assessment of coma and impaired consciousness; a practical scale. Lancet.

[40] Tuominen R, Joelsson P, Tenovuo O (2012). Treatment costs and productivity losses caused by traumatic brain injuries. Brain Inj.

[41] Turgeon AF, Lauzier F, Burns KE, Meade MO, Scales DC, Zarychanski R, Moore L, Zygun DA, McIntyre LA, Kanji S, Hebert PC, Murat V, Pagliarello G, Fergusson DA (2013). Determination of neurologic prognosis and clinical decision making in adult patients with severe traumatic brain injury: a survey of Canadian intensivists, neurosurgeons, and neurologists. Crit Care Med.

[42] van Dijck JT, Reith FC, van Erp IA, van Essen TA, Maas AI, Peul WC, de Ruiter GC (2018). Decision making in very severe traumatic brain injury (Glasgow Coma Scale 3-5): a literature review of acute neurosurgical management. J Neurosurg Sci.

[43] van Essen TA, de Ruiter GC, Kho K, Peul WC (2017). Neurosurgical treatment variation of traumatic brain injury: evaluation of acute subdural hematoma management in Belgium and the Netherlands. J Neurotrauma.

[44] van Essen TA, Dijkman MD, Cnossen MC, Moudrous W, Ardon H, Schoonman GG, Steyerberg EW, Peul WC, Lingsma HF, de Ruiter GCW (2018) Comparative effectiveness of surgery for traumatic acute subdural hematoma in an aging population. J Neurotrauma. 10.1089/neu.2018.5869 10.1089/neu.2018.5869 30234429

[45] von Steinbuchel N, Wilson L, Gibbons H, Hawthorne G, Hofer S, Schmidt S, Bullinger M, Maas A, Neugebauer E, Powell J, von Wild K, Zitnay G, Bakx W, Christensen A, Koskinen S, Formisano R, Saarajuri J, Sasse N, Truelle J (2010). Quality of life after brain injury (QOLIBRI): scale validity and correlates of quality of life. J Neurotrauma.

[46] von Steinbuchel N, Wilson L, Gibbons H, Hawthorne G, Hofer S, Schmidt S, Bullinger M, Maas A, Neugebauer E, Powell J, von Wild K, Zitnay G, Bakx W, Christensen AL, Koskinen S, Sarajuuri J, Formisano R, Sasse N, Truelle JL (2010). Quality of life after brain injury (QOLIBRI): scale development and metric properties. J Neurotrauma.

[47] Whitmore RG, Thawani JP, Grady MS, Levine JM, Sanborn MR, Stein SC (2012). Is aggressive treatment of traumatic brain injury cost-effective?. J Neurosurg.

[48] Wilson JT, Pettigrew LE, Teasdale GM (1998). Structured interviews for the Glasgow Outcome Scale and the extended Glasgow Outcome Scale: guidelines for their use. J Neurotrauma.

[49] Wilson L, Marsden-Loftus I, Koskinen S, Bakx W, Bullinger M, Formisano R, Maas A, Neugebauer E, Powell J, Sarajuuri J, Sasse N, von Steinbuechel N, von Wild K, Truelle JL (2017). Interpreting quality of life after brain injury scores: cross-walk with the short form-36. J Neurotrauma.

[50] Wilson WT, McMillan T, Young AM, White MA (2017). Increased trends in the use of treatment-limiting decisions in a regional neurosurgical unit. Br J Neurosurg.

