# Supplementary material for: Functional and patient-reported outcome versus in-hospital costs after traumatic acute subdural hematoma (t-ASDH): a neurosurgical paradox?
Source: Acta Neurochir (Wien). 2019 Mar 28;161(5):875–84. doi: 10.1007/s00701-019-03878-5 (PMC6483942; doi:10.1007/s00701-019-03878-5)
Supplement: Supplementary file 1 — (DOCX 21 kb) [file 701_2019_3878_MOESM1_ESM.docx]

Supplement 1

Functional and patient reported outcome versus in-hospital costs after traumatic acute subdural hematoma (t-ASDH); a neurosurgical paradox?

**Journal: Acta Neurochirurgica**

Jeroen T.J.M. van Dijck MD^1^, Thomas A. van Essen MD^1^, Mark D. Dijkman MD^1^, Cassidy Q.B. Mostert BSc^1^, Suzanne Polinder PhD^2^, Wilco C. Peul MD PhD MBA^1^, Godard C.W. de Ruiter MD PhD^1^
1. Neurosurgical Center Holland, Leiden University Medical Center & Haaglanden Medical Center & Haga Teaching Hospital, The Netherlands;
2. Department of Public Health, Erasmus Medical Center, The Netherlands

Corresponding author: Jeroen T.J.M. van Dijck, MD
Address: dept. of Neurosurgery LUMC & HMC, Albinusdreef 2, J-11-R-83 2333 ZA, Leiden & The Hague, Netherlands. Email: [j.t.j.m.van_dijck@lumc.nl](mailto:j.t.j.m.van_dijck@lumc.nl). Phone: +31715262109. Fax:+31715266987.

Supplement 1

| **Health care unit** | **Value (€)** | **Reference** | **Source** | **Value (2012)** | **Remark** |
| --- | --- | --- | --- | --- | --- |
| **Transportation:** |  | | | | |
| Ambulance | 613 | 2014 | Guideline^14^ | 592 | Costs for all immediate transportations. |
| Mobile Medical Team (MMT) involvement | 3,424 | 2011 | Multiple sources | 3,509 | Per unit. See * for calculation. |
| **Admission:** |  |  |  |  |  |
| ER visit | 259 | 2014 | Guideline^14^ | 250 | Per visit. |
| ICU admission | 2,257.27 | 2012 | NZA prices^29^ | 2,257 | Costs per day (category used: medium heavy). |
| High care admission | 659.84 | 2012 | NZA prices^29^ | 660 | Costs per day. |
| Ward admission (general/academic hospital) | 443/642 | 2014 | Guideline^14^ | 428/620 | Costs per day. |
| In-hospital consultations | 70-205 | 2012 | NZA prices (DIS)^28^ | 70-205 | Involvement of other specialists irrespective of reason/diagnosis. Counted as one visit. |
| Paramedical (such as physiotherapy, vocational therapy) | 30-33 | 2014 | Guideline^14^ | 29-32 | Price per visit. Involvement counted as 1. |
| Outpatient clinic visit (general/academic hospital) | 80/163 | 2014 | Guideline^14^ | 77/158 | Per visit, related to injury. |
| **Surgical intervention**** |  | | | | |
| - Craniotomy | 6,705 | 2012 | NZA prices (DIS)^28^ | 6,705 | Brain surgery, without admission costs. |
| - Decompressive craniectomy | 6,705 | 2012 | NZA prices (DIS)^28^ | 6,705 | Brain surgery, without admission costs. |
| - Replacement of bone flap | 2,795 | 2012 | NZA prices (DIS)^28^ | 2,795 | Brain surgery, without admission costs. |
| - External ventricular drainage | 4,830 | 2012 | NZA prices (DIS)^28^ | 4,830 | Brain surgery, without admission costs. |
| - Placement of ICP monitor | 2,795 | 2012 | NZA prices (DIS)^28^ | 2,795 | Brain surgery, without admission costs. |
| - Burr hole drainage | 2,475 | 2013 | NZA prices (DIS)^28^ | 2,415 | Brain surgery, without admission costs. |
| - Extracranial surgeries | 1,410-2,435 | 2012 | NZA prices (DIS)^28^ | 1,410-2,435 | Extracranial surgeries, without admission costs. |
| **Imaging studies** |  | | | | |
| - CT brain | 192.40 | 2012 | NZA prices^29^ | 192 | Per unit. |
| - CT spine | 192.40 | 2012 | NZA prices^29^ | 192 | Per unit. |
| - MRI brain | 238.32 | 2012 | NZA prices^29^ | 238 | Per unit. |
| - Other imaging studies | 48.61-296.96 | 2012 | NZA prices^29^ | 49-297 | Different studies, price per unit. |
| **Laboratory studies***** |  | | | | |
| -Arterial blood gas | 11 | 2012 | NZA prices^29^ | 11 | Per unit. |
| -Haematology | 10 | 2012 | NZA prices^29^ | 10 | Per unit. |
| -Chemistry | 50 | 2012 | NZA prices^29^ | 50 | Per unit. |
| -Coagulation | 20 | 2012 | NZA prices^29^ | 20 | Per unit. |
| -Blood culture | 31.94 | 2012 | NZA prices^29^ | 32 | Per unit. |
| -Other cultures (urine, CSF, sputum etc) | 13.74 | 2012 | NZA prices^29^ | 14 | Per unit. |
| **Blood products** |  |  |  |  |  |
| -Erythrocytes/thrombocytes/plasma | 216/522/186 | 2014 | Guideline^14^ | 209/504/180 | Per unit. |
| -Prothrombin complex | 210.94 | 2017 | medicijnkosten.nl | 199 | Per 10ml/250IE. If amount unknown: 20ml. |

**Caption:**

Supplement 1 shows a detailed overview of all used unit costs and corresponding sources.

**Legend:**

*Own calculation: National total costs for 2011: 21.3 million EUR (source: <https://www.recht.nl/rechtspraak/uitspraak/?ecli=ECLI:NL:CBB:2015:428>; accessed 2018 March 29). National total use: 6220 units (https://www.ambulancezorg.nl/static/upload/raw/1aa59bb7-a8e0-48e5-897e-06f9703a7e3d/ambulances-in-zicht-2015.pdf; accessed 2018 March 29). Unit price = €3,424 (21,300,000/6.223).
**Surgical interventions are classified according to the most frequently recorded operation in the database (www.opendisdata).
***Due to the major variety in laboratory studies only the most commonly performed studies were counted. Prices are calculated based on the NZA prices of individual studies.

**References**:

14. Hakkaart-van Roijen L, van der Linden N, Bouwmans C, Kanters T, Tan S (2015) Kostenhandleiding: methodologie van kostenonderzoek en referentieprijzen voor economische evaluaties in de gezondheidszorg. Zorginstituut Nederland Geactualiseerde versie 2015 https://www.zorginstituutnederland.nl/binaries/zinl/documenten/publicatie/2016/02/29/richtlijn-voor-het-uitvoeren-van-economische-evaluaties-in-de-gezondheidszorg/Richtlijn+voor+het+uitvoeren+van+economische+evaluaties+in+de+gezondheidszorg+%28verdiepingsmodules%29.pdf Accessed March 29 2018

29. Nederlandse Zorgautoriteit Tarieventabel DBC-zorgproducten en overige producten - per 1 januari 2012 (PUC_12710_22). <https://puc.overheid.nl/nza/doc/PUC_12710_22/1/>. Accessed March 29 2018

28. Nederlandse Zorgautoriteit. Open data van de Nederlandse Zorgautoriteit. http://<www.opendisdata.nl>. Accessed March 29 2018
